# Chondrocyte Homeostasis and Differentiation: Transcriptional Control and Signaling in Healthy and Osteoarthritic Conditions

**DOI:** 10.3390/life13071460

**Published:** 2023-06-28

**Authors:** Yara M. Michelacci, Raquel Y. A. Baccarin, Nubia N. P. Rodrigues

**Affiliations:** 1 Escola Paulista de Medicina, Universidade Federal de São Paulo, São Paulo 04023-062, SP, Brazil; 2 Faculdade de Medicina Veterinária e Zootecnia, Universidade São Paulo, São Paulo 05508-270, SP, Brazil; baccarin@usp.br (R.Y.A.B.); nubiarodrigues@usp.br (N.N.P.R.)

**Keywords:** chondrocyte transcription control, differentiation, apoptosis, osteoarthritis, chondroitin sulfate

## Abstract

Chondrocytes are the main cell type in articular cartilage. They are embedded in an avascular, abundant, and specialized extracellular matrix (ECM). Chondrocytes are responsible for the synthesis and turnover of the ECM, in which the major macromolecular components are collagen, proteoglycans, and non-collagen proteins. The crosstalk between chondrocytes and the ECM plays several relevant roles in the regulation of cell phenotype. Chondrocytes live in an avascular environment in healthy cartilage with a low oxygen supply. Although chondrocytes are adapted to anaerobic conditions, many of their metabolic functions are oxygen-dependent, and most cartilage oxygen is supplied by the synovial fluid. This review focuses on the transcription control and signaling responsible for chondrocyte differentiation, homeostasis, senescence, and cell death and the changes that occur in osteoarthritis. The effects of chondroitin sulfate and other molecules as anti-inflammatory agents are also approached and analyzed.

## 1. Cartilage Organization: Chondrocytes and Extracellular Matrix

Most of the volume of cartilage is an avascular extracellular matrix (ECM) in which chondrocytes are embedded. Collagen and proteoglycans constitute most ECM macromolecules, and together with several other proteins, they have been well characterized in recent years. 

The main cartilage collagen is type II, fibrillar, although many other fibril-forming, network-forming, FACITs, and microfibrillar collagens are also present. These minor collagens have different distribution patterns in the tissue, changing with development and disease. An excellent review of cartilage collagens appeared in 2017 [1]. Type VI collagen forms hexagonal networks close to cells where it can be linked to type II collagen fibrils via biglycan and matrilin-4 [2]. Other collagens, such as types IX, XII, XIV, and XXII, are associated with the surfaces of fibrils, such as type IX collagen, which contains a chondroitin sulfate chain linked to the globular domain 2 of the alpha 2(IX) chain [3]. Type X collagen is synthesized by hypertrophic chondrocytes and is present in hypertrophic cartilage and calcified zones [4].

The major non-collagenous macromolecules found in articular cartilage are proteoglycans, glycoconjugates formed by a protein core with at least one covalently linked glycosaminoglycan side chain [5,6,7,8]. The main cartilage proteoglycan is aggrecan, which belongs to the hyalectan family. Versican, perlecan, and the small leucine-rich proteoglycans (SLRPs) decorin, biglycan, fibromodulin, lumican, chondroadherin, and proline/arginine-rich end leucine-rich repeat protein (PRELP) are also present, performing different functions [9]. 

Some other proteins, such as cartilage oligomeric matrix protein (COMP), matrilins, chondromodulin, and pleiotrophin, whose functions range from involvement in ECM organization to the modulation of the chondrocyte phenotype, have also been described [10]. Changes occur in the cartilage ECM under physiological conditions, such as embryonic development and growth, and pathological conditions, such as osteoarthritis (OA) [11].

**Figure 1:**
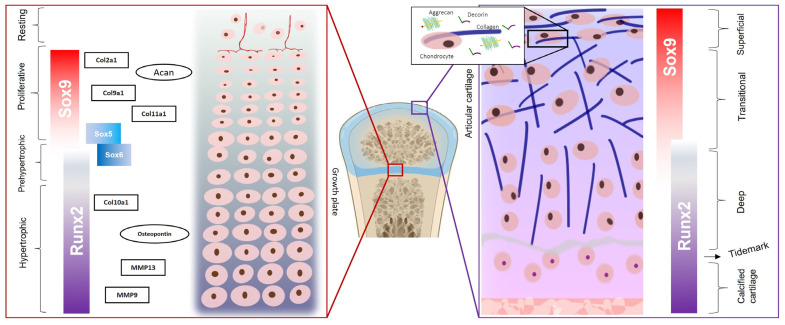
Schematic representation of articular cartilage and growth plate chondrocytes, with phase-specific master transcription factors. SOX9 (SRY-box-containing protein 9) induces the expression of cartilage-ECM-coding genes collagen type 2 (*col2a1*), collagen type IX (*col9a1*), collagen type XI (*col11a1*), and aggrecan (*Acan*), while RUNX2 (Runt-related transcription factor 2) activates the expression of collagen type X (*col10a1*), osteopontin, and metalloproteinases (MMP-9 and MMP-13).

Healthy articular cartilage contains four regions that differ in their relative amounts of collagen fibrils and proteoglycans, as well as in their dispositions: (a) the superficial zone, with low concentrations of aggrecan and high levels of decorin; (b) the transitional zone; (c) the deep zone; (d) the calcified zone, immediately above the subchondral bone and below the “tidemark”, which is a calcified zone that persists after the growth plate closure. The cell density decreases from the superficial zone to the deep zone, while the cell volume and the proportions between the proteoglycans and collagen increase [12] (Figure 1).

During embryonic development, the skeletal elements of the axial and appendicular skeletons originate from the mesoderm and undergo endochondral ossification. The central mesodermal cells differentiate first into proliferating chondrocytes and then into pre-hypertrophic and hypertrophic chondrocytes, followed by the formation of mineralized cartilage. The growth plate is responsible for longitudinal bone growth. In mammals, it appears during pregnancy and fuses when growth terminates, consisting of distinct zones which reflect cell differentiation: (1) the resting zone, located at the top of the growth plate; (2) the proliferating zone in which the cells flatten and divide; (3) the pre-hypertrophic zone; (4) and the hypertrophic zone in which the chondrocytes are organized in columns and the cells become 5–12 times larger [13,14] (Figure 1).

Proliferating chondrocytes strongly express cartilage ECM genes, including aggrecan (*Acan*), collagen type II alpha 1 chain (*Col2a1*), collagen type IX alpha 1 chain (*Col9a1*), and collagen type XI alpha 1 chain (*Col11a1*), while hypertrophic chondrocytes specifically express collagen type X alpha 1 chain (*Col10a1*), osteopontin, and matrix metalloproteinases-9 and -13 (MMP-9 and MMP-13). The expression of these genes is controlled by master transcription factors that direct the genetic program of the differentiation of mesenchymal cells into chondrocytes and then the differentiation of proliferating chondrocytes [15] into hypertrophic chondrocytes [16].

Furthermore, the composition of the ECM differs in the territorial zone—closer to the cells—from the interterritorial zone [11]. The interterritorial zone is richer in fibrillar collagen, primarily type II collagen fibrils with types XI and IX collagens integrated, while the territorial matrix is richer in type VI collagen microfibrils and proteoglycans, with lower amounts of fibrillar collagen [17]. Collagen fibrils provide tensile strength throughout the ECM, while aggrecan plays a key role in controlling the compressive properties of cartilage.

## 2. Chondrocyte Differentiation

### 2.1. Control of Gene Expression

**Figure 2:**
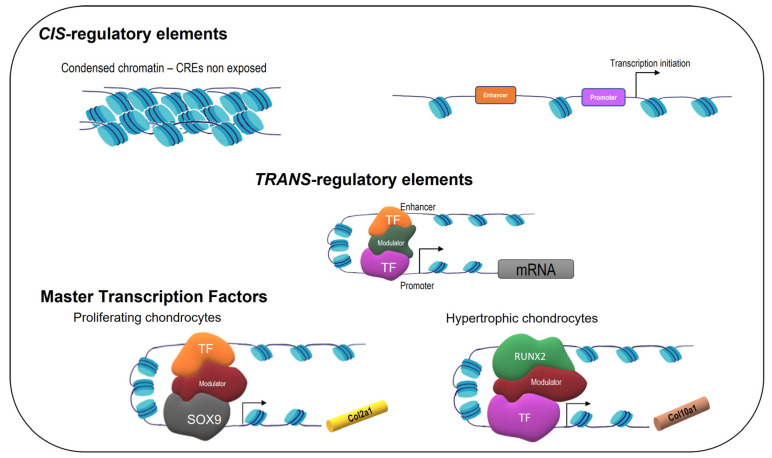
Overview of models of multi-layered gene regulatory mechanisms in chondrocytes.

Gene transcription is primarily determined by the interactions between *cis*-regulatory elements (CREs) and *trans*-regulatory factors. CREs are regions of non-coding DNA that contain binding sites for proteins that act as regulatory molecules. Promoters and enhancers are different types of CREs: promoters recruit the transcription machinery to initiate transcription, while enhancers control the temporal regulation of gene transcription. *Trans*-regulatory factors include transcription factors (TFs) and mediators [18]. The TFs bind to promoters and enhancers in a sequence-specific fashion, and the mediators bind to the TFs. However, the real situation is much more complicated because CREs may or may not be exposed to TFs, and these form a transcriptional complex with mediators and other TFs controlling the chromatin state in a hierarchic fashion (Figure 2).

Transcriptional activation or repression requires the recruitment of chromatin remodeling complexes that control the higher-order structure of chromatin, facilitating or hindering the binding of TFs and aiding or preventing the organization of the transcription preinitiation complex. There are two types of chromatin remodeling complexes that are recruited by transcription activators and repressors: ATP-dependent chromatin remodeling complexes and histone-modifying enzymes (histone deacetylases, histone acetyltransferases, and histone kinases) [19]. Furthermore, DNA methylation must be considered [20]. There is evidence that changes in epigenetics may affect chondrocyte homeostasis, leading to OA [21].

Concerning CREs, the mouse *Col2a1* intron 1 enhancer was shown to be sufficient for the determination of chondrocyte differentiation [22]. The *Col10a1* enhancer was also identified as specific to hypertrophic chondrocytes [23]. Regarding the *trans*-regulatory factors, there are a limited number of master regulators identified. The SRY-box-containing gene 9 (SOX9) binds to the *Col2a1* enhancers in chondrocytes [16], while Runt-related transcription factor 2 (RUNX2) binds to the *Col10a1* enhancers in chondrocytes and to the *osteocalcin-specific element 2* (*OSE2*) in osteoblasts [24]. RUNX2 is necessary for both chondrocyte hypertrophy and osteoblast specification. 

By using an assay that allows for access to the chromatin-accessible regions (ChIP-Seq, chromatin immunoprecipitation, and sequencing), it was shown that the *SOX9* motif appeared as open chromatin in resting and proliferating chondrocytes, whereas the *RUNX* motif appeared in hypertrophic chondrocytes, suggesting that different CRE repertories are available in different cell types [10,18].

Homeostatic mechanisms respond to the mechanical load and strain to which articular cartilage and bone are exposed under normal conditions, [25], but eventually these mechanisms are not sufficient, and the cartilage loss that is typical of OA begins. During the initial period, increased proliferation and enhanced tissue remodeling occur, with the synthesis of new cartilage ECM components [26,27]. Eventually, the secretion of pro-inflammatory cytokines (including interleukin-1β, IL-1β), MMPs, and a disintegrin and metalloproteinase with thrombospondin motifs (ADAMTSs) [28] begins. IL-1β creates an inflammation microenvironment that leads to the loss of ECM components, chondrocyte hypertrophy, and terminal differentiation. Chondrocytes begin to express RUNX2, vascular endothelial growth factor (VEGF), collagen X, and MMP-13 [28,29]. This is a shift towards hypertrophy, followed by the calcification of the ECM around chondrocytes and subchondral bone sclerosis. These changes in both cartilage and bone occur before the onset of clinical symptoms.

### 2.2. SOX9, SOX5, and SOX6

*SOX9* is an essential cartilage-promoting factor. It is expressed early in mesenchymal condensations throughout the embryo. It was shown that *SOX9^−/−^* embryonic stem cells did not express chondrocyte-specific markers such as *Acan*, *Col2a1*, *Col9a2*, and *Col11a2* and did not form any cartilage [30]. In vivo studies performed by different groups have shown that *SOX9* inactivation in differentiated chondrocytes leads to decreased proliferation, increased death, and the dedifferentiation of chondrocytes in the growth plate, as well as to aggrecan degradation in articular cartilage [31,32]. The expression of *SOX9* decreases in monolayer cultured chondrocytes with passages, but upon transduction with retroviral *SOX9*, these chondrocytes regained a chondrocyte phenotype and were able to form a cartilaginous ECM [33]. *SOX9* is expressed in proliferating chondrocytes but is downregulated when chondrocytes reach hypertrophy (Figure 1).

SOX9 contains a potent transactivation domain that permits the recruitment of transcriptional co-activators, including components of the general transcriptional machinery, mediator complex, and histone-modifying enzymes. SOX9 is frequently described in conjunction with two other transcription factors that belong to the SRY-box-containing proteins: SOX5 (originally called L-SOX5 or SOX5L) and SOX6. They are sometimes collectively called “the SOX trio” or “chondrogenesis trio”. SOX5 and SOX6 belong to the SOXD group [34], while SOX9 belongs to the SOXE group [35].

SOX5 and SOX6 do not contain transactivation domains but, like SOX9, they are expressed in early chondrocytes [36], and their expression is abruptly turned off in chondrocyte hypertrophy. SOX5 and SOX6 are necessary for efficient chondrogenesis. The inactivation of either *SOX5* or *SOX6* leads to mild defects in the genesis of the skeleton, but the co-inactivation of the two genes results in the underdevelopment of the growth plate and articular cartilage with the production of matrix-deficient cartilage [37], suggesting that *SOX5* and *SOX6* may be redundant genes.

### 2.3. GLI1, GLI2, and GLI3

**Figure 3:**
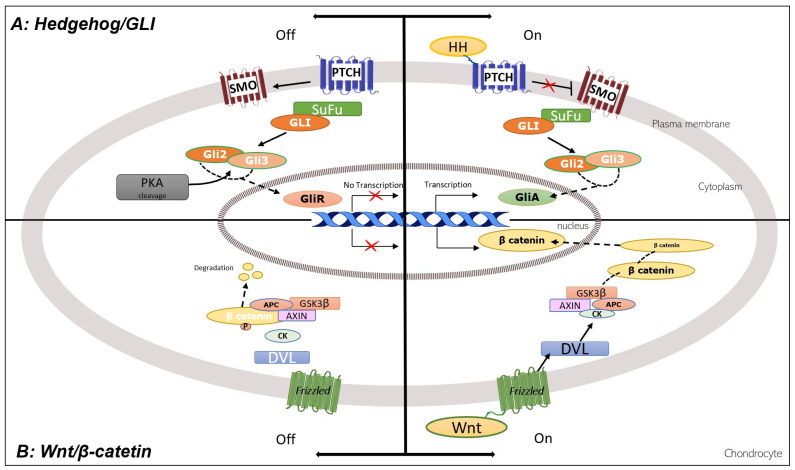
Hedgehog/GLI and Wnt/β-catenin signaling pathways in chondrocytes. See text for abbreviations [38].

An important family of zinc-finger TFs is named GLI, including GLI1, GLI2, and GLI3 [38]. GLI1 functions as a strong transcription activator, and GLI2 and GLI3 function both as full-length activators (GliAs) and truncated repressors (GliRs) (Figure 3).

The transition from the repressor form to the activator form is controlled by the hedgehog (HH) proteins, better described in Section 3.1. *GLI1*, *GLI2*, and *GLI3* are essential for chondrocyte differentiation and are expressed in proliferating chondrocytes. *GLI3* expression is turned off in pre-hypertrophic chondrocytes, while *GLI2* expression decreases in hypertrophic chondrocytes.

### 2.4. RUNX2

RUNX2 is a TF essential for chondrocyte hypertrophy and for osteoblast differentiation [39]. *RUNX2* is weakly expressed in proliferating chondrocytes, but its expression increases in chondrocytes that exit the cell cycle. It was shown that the expression of *RUNX2* in proliferating chondrocytes accelerates hypertrophy [40], whereas the removal of *RUNX2* stops normal hypertrophic cartilage mineralization.

RUNX2 binds to the *Col10a1* enhancer in chondrocytes (Figure 2) and at the *osteocalcin-specific element 2* (*OSE2*) in osteoblasts [24]. In the chromatin of *Col10*-positive chondrocytes, the RUNX2-binding regions are accessible. RUNX2 binds to DNA both near and distal to the transcription start site, and recent data suggest that RUNX2 is involved in higher-order chromatin organization [41]. Furthermore, in a model of OA, it was recently reported that RUNX2-DNA binding regions are changed by inflammation [42].

### 2.5. ncRNAs: miRNAs, lncRNAs, and circRNA

Non-coding RNAs (ncRNAs) are a large segment of the transcriptome that do not appear to play any protein-coding functions but may be involved in many biological processes, including diseases.

#### 2.5.1. miRNAs

**Figure 4:**
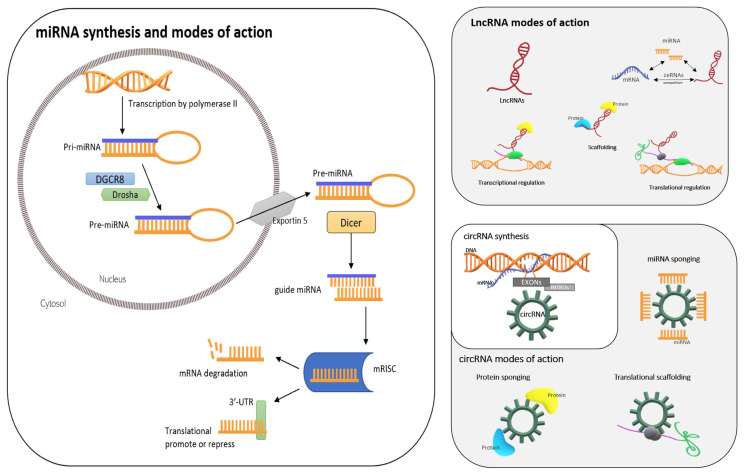
Non-coding RNAs: micro RNAs (miRNAs), long non-coding RNAs (lncRNAs), and circular RNAs (circRNAs). The Figure shows their origins and mechanisms of action.

Micro RNAs (miRNAs or miRs) are ~22-nucleotide-long ncRNAs that directly bind to target RNAs in a sequence-complementary manner and facilitate degradation or inhibit the translation of their target messenger RNAs (mRNAs). miRNAs are transcribed by RNA polymerase II—the same enzyme that transcribes mRNAs—as long transcripts named primary microRNA transcripts (pri-miRNAs). These are subsequently processed by an enzyme complex formed by RNase III, Drosha, and DGCR8 into small hairpin precursor miRNAs (pre-miRNAs) (Figure 4), which are transported to the cytoplasm via exportin 5 to be further cleaved by the enzyme Dicer at the stem loop to generate a ~22-nucleotide imperfect double-stranded miRNA duplex [43]. One strand of this duplex, known as the guide miRNA, remains associated with the miRNA-induced silencing complex (mRISC), while the other strand is rapidly degraded. This miRNA guides the mRISC to the 3′-UTR of its target mRNA, which is partially or fully complementary to the sequence of the miRNA, and this binding to the 3′-UTR can promote or repress the translation of the target mRNA [44] (Figure 4).

Changes in the expression of miRs may induce OA [45]. Several miRs that participate in chondrogenesis have been identified—some upregulated and some downregulated [46]. Some miRs mediate joint-protective mechanisms, while others participate in processes that contribute to OA. In 2018, Endisha et al. published a very comprehensive review of this issue [47]. Some miRs, such as miR-320a, miR-441, and miR-381, target MMPs, while others, such as miR-140, miR-30a, and miR-148a, target ADAMTSs, enzymes that breakdown aggrecan. Some miRs regulate inflammatory pathways, some regulate cellular apoptosis mechanisms, and others regulate other biological pathways.

For instance, miR-140 targets a co-repressor of RUNX2, which is essential for chondrocyte hypertrophy [48], and ADAMTS5 [49]. Its expression is greatly reduced in OA [48] and is suppressed by IL-1β signaling, while its overexpression protects against OA [49]. Additionally, miR-27a and miR-27b target MMP-13 expression, and miR-27b expression is suppressed by NFκB signaling and mitogen-activated protein kinase (MAPK) [20].

Recently, miR-146a-5p was identified as the most responsive miR to the proinflammatory cytokine IL-1β in primary murine chondrocytes [50]. miR-146a-5p was upregulated in human OA cartilage, and the knockdown of miR-146a-5p antagonized IL-1β-mediated inflammatory and catabolic responses in vitro. Moreover, the silencing of *miR-146a-5p* in chondrocytes ameliorated articular cartilage destruction and reduced OA symptoms in a murine OA model. The authors suggested that miR-146a-5p could be a potential therapeutic target in treating OA [50].

#### 2.5.2. lncRNAs

Long non-coding RNAs (lncRNAs) are RNAs > 200 nucleotides long, with little or no protein-coding potential, that are transcribed by RNA polymerase II [51]. They can fold into unique conformations that permit interactions with DNA, RNA, and proteins, enabling them to participate in many regulatory networks [52], influencing gene expression through multiple mechanisms such as chromatin remodeling, competing endogenous RNAs (ceRNAs), mRNA stabilization, and the recruitment of scaffolding proteins. One important mechanism of action of lncRNAs is their regulation of higher-order chromatin structure through interactions with miRNAs and remodeler complexes [53] (Figure 4).

There is increasing evidence that lncRNAs are involved in the regulatory mechanisms of chondrogenesis and osteogenesis as well as OA. For instance, lncRNA-HIT exists in E11 mouse embryos and forms lncRNA-HIT-p100/CBP regulatory complexes during initial chondrogenic differentiation [54], which is essential for mesenchymal cell condensation and the formation of cartilage nodules. Therefore, lncRNA-HIT is necessary for mesenchymal chondrogenic differentiation.

Another lncRNA, lncRNA LOC102723505, which is also called the regulator of chondrogenesis RNA (ROCR), is upregulated in chondrogenic differentiation [55]. The lncRNA ROCR contributes to the expression of SOX9 and is necessary for matrix glycosaminoglycan production [56]. The depletion of ROCR impacts SOX9 expression and the production of matrix components, which ultimately disrupts mesenchymal cell chondrogenesis [52]. 

In OA, chondrocyte apoptosis, the degradation of cartilage ECM, and aberrant changes in subchondral bones, inflammatory responses, and angiogenesis occur. Recent studies on the expression of lncRNAs suggest correlations between lncRNAs and OA (review in [52]). Some lncRNAs interfere directly or indirectly with ECM degradation, chondrocyte proliferation or apoptosis, inflammatory processes, or angiogenesis. Their targets may be proteins, such as ADAMTS5, VEGF, or Wnt3a factors, and many different miRNAs [52].

#### 2.5.3. circRNAs

Circular RNAs (circRNAs) are closed-loop, non-coding RNAs that play important regulatory roles in transcription and translation. They are produced via the reverse splicing of a precursor mRNA [57] (Figure 4). Most circRNAs are single-stranded RNAs composed of single or multiple exons [58]. They are highly stable, conserved, and tissue-specific [59] (Figure 4).

Although most circRNAs are localized in the cytoplasm, some are present in the nucleus. One example is ci-ankrd52, which binds RNA polymerase II to promote gene transcription; other examples are CircEIF3J and CircPAIP2, which bind to the U-small ribonucleoprotein and then to RNA polymerase II to similarly regulate gene expression [60]. circRNAs seem to participate in OA by combining mechanisms of oxidative and mechanical stress, autophagy, proliferation, and apoptosis. Zhang et al. recently published an excellent and comprehensive review on circRNAs in OA [61].

## 3. Signaling in Chondrocyte

Chondrogenesis is regulated by complex and integrated signaling pathways, including the wingless-type (Wnt)/β-catenin [62], hedgehog (HH)/GLI [63], bone morphogenetic protein (BMP)/SMAD4 [64], fibroblast growth factor (FGF) [65], and Notch/nuclear factor-κB (NFκB) pathways [66].

### 3.1. Hedgehog, GLI, TRPSI, and Wnt/Catenin

An important family of signaling proteins that control gene expression during skeletal development is named hedgehog (HH). There are three HH genes in mammals: Sonic hedgehog (SHH), Indian hedgehog (IHH), and Desert hedgehog (DHH). 

HHs control the transcription of specific genes through the zinc-finger TFs GLI1, GLI2, and GLI3 [38]. A protein named suppressor of fused (SuFu) complexes with GLI factors and keeps them in the cytoplasm [67]. Without HH input, GLI2 and GLI3 are phosphorylated and then cleaved to generate the transcriptional repressor forms (GLI repressors: GliRs) that repress the transcription of target genes (Figure 3).

The receptor of HH is a twelve-pass transmembrane protein named patched (PTCH). Without HH ligands, PTCH inhibits smoothened (SMO), a seven-pass transmembrane protein that has an intrinsic intracellular signaling activity. The binding of HH to PTCH on the target cell relieves the repression of PTCH on SMO [68], and the released SMO prevents the proteolytic processing of GLI2 and GLI3. Thus, the GLI activator forms translocate into the nucleus to activate the transcription of target genes while derepressing another set of genes.

Tricho-rhino-phalangeal syndrome 1 (TRPS1), a gene involved in an autosomal dominant skeletal disorder, is a TF with nine zinc-finger motifs. TRPS1 has many functions in cartilage and other tissues. TRPS1 regulates the differentiation, proliferation, and apoptosis of chondrocytes in the growth plate through interactions with several signaling molecules [69].

Wingless type (Wnt) is a family of 19 secreted glycoproteins that participate in development and homeostasis signaling in different tissues, including bone and cartilage (reviewed in [70]). Wnts bind to receptors of the *frizzled* (FZD) family and initiate several distinct pathways. The best-known pathway involves the β-catenin nuclear effector. In the absence of Wnt, β-catenin is phosphorylated and degraded in proteasome. The binding of Wnt to FZD activates a protein named dishevelled (DVL) which inhibits β-catenin phosphorylation and degradation. β-catenin translocates to the nucleus and controls the transcription of target genes [71] (Figure 3).

The differentiation of the proliferating into hypertrophic chondrocytes is a key step that determines the length of long bones. The expression of different signaling molecules, among them BMPs, IHH, and Wnt, is controlled by master TFs. It was recently shown that the TF TRSP1 and the activator form of GLI3 (GLI3A) activate the expression of Wnt5a via binding to upstream regulatory sequences in the Wnt5a promoter, identifying Wnt5a as a target gene for TRSP1 and GLI3A in chondrocytes [72].

### 3.2. TGFβ, BMP, and SMAD4

The TGF-β superfamily consists of TGF-βs, BMPs, and other related proteins. These proteins act through cell surface receptors that transduce intracellular signals via the SMAD complex or MAPK pathway.

TGF-βs and BMPs are important in many steps of skeleton development [73]. They are also very important in cartilage and bone homeostasis in adults, as well as in fracture healing. There is crosstalk between TGF-β/BMP signaling and other critical signaling pathways such as Wnt, Hedgehog, Notch, and FGF to coordinate these processes. Excellent reviews on this topic were recently published [29,74].

### 3.3. FGF

Fibroblast growth factors (FGFs) comprise a family of secreted proteins that bind and activate their cognate receptors (FGFRs). To date, 24 members of this family have been identified. They are highly expressed during embryonic development, and some of them—FGF1, 2, 8, 9, and 18—seem to participate in OA.

The binding of FGFs to the extracellular domain of FGFRs induces the phosphorylation of the tyrosine residues of the FGRSs’ intracellular domain. Phosphorylated FGFRs recruit target proteins and activate the downstream signaling pathway [75,76].

It seems that FGF1, FGF2, and FGF8 have catabolic effects on chondrocytes and articular cartilage. FGF1 is expressed in chondrocytes isolated from OA patients [77], and the repression of MMP-13 was observed in FGF1-treated human chondrocytes [78]. FGF2 acts through RUNX2 and ADAMTS5 activation [79], and its levels positively correlate with MMP-1 and MMP-13 but negatively correlate with *Acan* and *Col2a1* expression in human OA chondrocytes [80]. FGF8 caused an increase in the levels of MMP-3 and prostaglandin E2 (PGE2), which causes degradation of the ECM [81].

FGF9, in contrast, seems to be vital in skeletogenesis and joint formation. It is highly expressed by many cell types, including chondrocytes of the growth plate. In comparison to healthy articular cartilage, FGF9 is significantly decreased in the articular cartilage of OA patients, and an intra-articular injection of recombinant FGF9 reduced cartilage degeneration and down-regulated the biomarkers of chondrocyte hypertrophy, collagen X and MMP-13 [82]. These data suggest that FGF9 has a protective effect on damaged articular cartilage.

FGF18 has significant anabolic effects on cartilage [83]. It was shown that the administration of FGF18 induced dose-dependent increases in cartilage thickness in an OA model in rats [84], prevented cartilage degeneration, and stimulated the repair of damaged cartilage [85]. A clinical study of FGF18 recombinant protein (Sprifermin, recombinant human FGF18, rhFGF18) in OA treatment is ongoing [86].

### 3.4. Notch

Signaling between adjacent cells via the Notch receptor regulates cell fate during animal development. Notch is a single-pass transmembrane protein which, when activated via binding to specific ligand on the cell surface of another cell, is cleaved by a membrane-bound protease, and the cytoplasmic tail translocates into the nucleus to bind to a transcription repressor which, upon binding, becomes a transcription activator.

It was shown that in articular cartilage chondrocytes, as the cells are not in close contact with each other, the activation of Notch signaling may be associated with the development of OA [87]. Notch receptors are highly expressed and are located at the cell surfaces of chondrocytes in articular cartilage, but the overexpression of the Notch intracellular domain leads to an increased expression of MMP-13 [87].

### 3.5. NFkB, TLR, TNF-α, and IL-1

NFκB is a family of latent gene regulatory proteins that respond to infection or injury. An inappropriate inflammatory response may lead to pain, tissue damage, and even cancer. In mammals, there are five NFκB proteins that form homo- and heterodimers. These dimers bind to inhibitory proteins named IκB that hold them in the cytoplasm in an inactive state. When this pathway is activated, IκB is phosphorylated and degraded, and NFκB is activated and goes into the nucleus. TNFα and IL-1, which are cytokines that are particularly important in inducing inflammatory responses, and the Toll-like receptors (TLRs) activate the NFκB pathway [88,89].

## 4. Chondrocyte Death

### 4.1. Necrosis, Apoptosis, Chondroptosis, and Autophagy

Cartilage damage and chondrocyte loss occur with aging [90] and after joint injury [91] or inflammatory diseases. Joint injuries are common among young athletes, frequently leading to the early onset of OA. In athletic horses, a similar OA is also observed, leading to the early retirement of these animals [11]. There is a correlation between chondrocyte death and ECM degradation, contributing to the chronic process that characterizes OA. 

Necrosis is a non-programmed and pathological form of cell death [92]. Chondrocytes may die via necrosis or by necroptosis, which is a programmed form of necrosis, or inflammatory cell death upon exposure to chemical or mechanical injury, inflammation, or infection [93]. Dead chondrocytes release intracellular components, generating reactive oxygen species (ROS, see next section) and inflammatory mediators, [94]. Both necrosis and necroptosis in chondrocytes may induce cartilage degradation [95]. Necroptotic and apoptotic cell deaths seem to have a common origin, but in necroptosis, a disruption of the membrane occurs, leading to an inflammatory reaction. 

In contrast, apoptosis, or programmed cell death, is a regulated pathway that involves specific intracellular signals and genes. An important feature of apoptosis is the condensation of chromatin and the cleavage of nuclear DNA into a characteristic ladder pattern of fragments. Two classical mechanisms leading to apoptosis were described: the intrinsic pathway, induced by intracellular signals, and the extrinsic or death receptor pathway, triggered by extracellular signals [96]. Both pathways require enzymes named caspases for their actions. In mammals, 14 caspases have been identified, which are classified into initiator caspases (caspases-2, -8, -9, and -10), effector caspases (caspases-3, -6, and -7), and inflammatory caspases (caspases-1, -4, -5, -11, -12, and -13). Caspase-14 is required for the cornification that plays a crucial role in the formation of the skin barrier.

Each apoptosis pathway uses its own caspases, which are activated by different complexes, and both pathways are tightly regulated by pro-apoptotic and anti-apoptotic proteins to ensure that the cells kill themselves only when it benefits the organism. Reviews on chondrocyte apoptosis have been published previously [97,98].

In contrast to necrosis, the uncontrolled release of intracellular components, particularly lysosomal enzymes, does not occur in apoptosis. Tissue-resident macrophages and other cells phagocyte the apoptotic bodies, but these cells are not present in cartilage, suggesting another mechanism to eliminate cell remnants.

The term chondroptosis [99] was created in 2004 to describe a type of cell death observed in chondrocytes: a special type of apoptosis in which the phagocytosis of cell remnants is difficult. Chondroptosis presents some characteristics in common with classical apoptosis: cellular shrinkage and chromatin condensation. However, in contrast to classical apoptosis, the rough endoplasmic reticulum is increased and expanded, and small patches of condensed chromatin spread throughout the nucleus. The most distinguishing features of chondroptosis are the autophagic vacuoles, which are responsible for the extrusion of cellular material into the extracellular space, suggesting that chondroptosis could be a combination of classical apoptosis and autophagy [89].

Autophagy is a cellular homeostatic mechanism which, through the formation of an autophagosome, plays an important role in the catabolic process and energy recycling of eukaryotic cells [100]. An autophagosome contains unwanted cell components and dysfunctional organelles and fuses with lysosomes to degrade these products. Autophagy is induced by different stimuli and participates in the regulation of cell death and inflammation [101]. A major negative regulator of autophagy is the mammalian target of rapamycin (mTOR), a serine/threonine protein kinase. Akt directly phosphorylates and activates mTOR, while mTOR negatively regulates phosphatidyl inositol-3-kinase (PI3K)/Akt activity and activates protein synthesis and ribosome biogenesis [102]. There is crosstalk between autophagy and apoptosis which is initiated by shared upstream signals [103]. There is evidence that autophagy is downregulated in OA, allowing for chondrocyte death and tissue destruction [104].

Finally, the relationship between extracellular vesicles, particularly articular cartilage vesicles (ACVs), autophagy, and apoptosis is considered in both normal chondrocytes and in OA. Extracellular vesicles are small, membrane-bound particles released from cells. They can be found in tissues, synovium, and blood and can be broadly classified into three categories: (1) microvesicles, produced by budding from the plasma membrane or fission, with a diameter of 100–1000 nm; (2) exosomes, produced through the fusion of multivesicular bodies, with a diameter less than 100 nm; and (3) apoptotic bodies, vesicles released during the process of apoptosis [105].

Extracellular vesicles are present in the pericellular matrix of articular cartilage and the growth plate. Regardless of their origin, these vesicles have the capacity to mineralize the matrix [106]. There are distinctive features between ACVs from normal individuals and from OA patients: immunoglobulins and complement components were present only in OA ACVs, which also contained lower levels of matrix proteoglycans [106]. Nevertheless, the relationship between apoptosis and ACVs is unclear. Although membrane blebbing is an early phenomenon in apoptosis [107], an increase in apoptotic cells does not lead to an increase in the number of ACVs, and ACVs isolated from apoptotic cells do not increase the degree of calcification [108]. ACVs are different from apoptotic bodies, which are much larger [109]. However, growing evidence suggests that autophagy is related to ACV generation [110], and a lack of autophagy may contribute to the development of OA [104,111].

### 4.2. Oxidative Stress-Induced Senescence

Many studies have emphasized the central role of senescent chondrocytes in the development and progression of OA. Some of them have shown that the proportion and distribution of senescent chondrocytes were positively correlated with the degree of joint injuries and the severity of OA [112]. However, it is important to emphasize that the senescent microenvironment in the OA joint includes not only senescent chondrocytes but also synovial fibroblasts and synovial macrophages [113]. The crosstalk between synovial cells and senesce may induce the development of OA by amplifying the inflammatory response and/or by prompting the senescence-associated secretory phenotype (SASP) [114].

Cellular senescence is a cell state characterized by irreversible cell cycle arrest, resistance to apoptosis, and the continuous secretion of SASP factors. The components of the SASP are cell type-dependent and vary with the inducer and duration of senescence and the surrounding microenvironment [115]. In OA, these factors include cytokines (IL-1, -6, and -8), chemokines, and enzymes (MMP-1, -3, -12, and -13) which exacerbate inflammation and promote the degradation of the ECM [113,116] and growth factors such as insulin-like growth factor binding proteins (IGFBP)-3, -4, and -7 that are involved in mediating senescence via paracrine signaling [114,115].

The SASP factors may induce senescence in neighbor chondrocytes through cell–cell communication, promoting OA progression [116]. Different pathways such as p38MAPK, JAK2/STAT3, inflammasome, mTOR, the PI3K pathway, HSP90, ncRNAs, GATA4/p62-mediated autophagy, macroH2A1, and ATM are all involved in the development and regulation of SASP. However, most of these cascades involved in inducing and regulating SASP converge on the activation of two transcription factors: NFκB and CCAAT/enhancer-binding protein-β (C/EBPβ) [115]. Thus, SASP factors can spread senescence by acting on senescent cells as well as on neighboring cells through autocrine and paracrine mechanisms.

The signaling mechanisms leading to senescence are numerous and complicated, but two main types of chondrocyte senescence were described: replicative senescence or intrinsic senescence and premature senescence or extrinsic senescence [114,117]. Replicative senescence is associated with changes in DNA structure and function and refers to reduced replicative capacity caused by telomere shortening and dysfunction after too many cell divisions. However, telomere loss does not completely explain senescence in non-replicating cells such as chondrocytes. So, it seems that extrinsic or stress-induced senescence is more relevant in articular cartilage [117].

Stress-induced premature *senescence* can occur in response to different damaging stimuli such as DNA damage, oxidative stress, chemotherapy, mitochondrial dysfunction, oncogenesis, epigenetic and chromatin perturbations, paracrine senescence (SASP profile), ionizing/ultraviolet radiations, and an altered translation process [116,118]. It is of note that most senescence-inducing stimuli have been associated with increases in reactive oxygen species/reactive nitrogen species (ROS/RNS) [119]. In OA, mechanical overloading, chronic inflammation, chemical toxicants, and an accumulation of oxidative products may contribute to chondrocyte senescence [116,120]. Of course, aging chondrocytes may undergo both replicative and premature senescence. 

Oxidative stress is considered a major contributor to DNA damage and cellular senescence and may play a major role in the link between aging and OA [120]. Oxidative stress occurs when the overproduction of ROS/RNS exceeds the capacity of available antioxidants. ROS/RNS are generated due to the aerobic metabolism, produced by specific enzymes, and following extracellular stimuli [119]. The main ROS/RNS produced by chondrocytes are NO and O^2−^, which generate NO_3_^−^ and H_2_O_2_ [121]. 

In a recent review [119], the major pathways inducing senescence via ROS/RNS deregulation were summarized. Evidence suggests that ROS and RNS can trigger senescence through different mechanisms: (1) NFκB stimulation and the induction of transcription of the main factors composing the SASP; (2) DNA double-strand breakages, triggering a sustained DNA damage response (DDR); (3) telomere shortening; (4) double cross-talk between mitochondria dysfunction and ROS/RNS production; and (5) the inhibition of nuclear factor erythroid 2–related factor 2 (Nrf2), an important antioxidant transcription factor [118].

However, the mechanisms through which oxidative stress triggers chondrocyte senescence are not well understood. Studies with different cells have suggested that the activity of MAP kinase pathways, which include ERK, JNK, and p38, may be particularly important in the induction of chondrocyte senescence [120]. Oxidative stress also induces cell senescence by activating PI3K/protein kinase B (PKB) signaling pathways [114]. Thus, excessive levels of ROS inhibits matrix synthesis by suppressing the IRS-1-PI3K-Akt signaling pathway or by activating the ERK/MAPK signaling pathway. The sustained activation of ERK can promote cell senescence. In addition, extracellular ROS could also add to the inhibition of the Akt pathway through oxidized low-density lipoprotein (LDL). Chondrocyte senescence can be induced via binding oxidized LDL to the cell surface receptor LOX-1 and, consequently, the inhibition of Akt phosphorylation upon IGF-1 stimulation [117].

The overproduction of ROS/RNS or ineffective antioxidant defense systems lead to oxidative damages to biomolecules, causing alterations to specific functions, signaling, and metabolic pathways [119] and leading to chondrocyte senescence [120]. Similar to what is seen in replicative senescence, ROS/RNS also induce irreversible single-strand DNA breaks at telomeres, also called the DNA damage response (DDR) [119]. A persistent DDR activates the p53/p21CIP1 pathway, while epigenetic changes due to the downregulation of the BMI-1 and EZH2 proteins of the PCRs complex induce the p16INK4a/Rb pathway and establish the SASP profile [119]. Both pathways are also interlinked with extensive crosstalk [115]. Although these two pathways share several common regulatory factors, p16/pRB pathway-induced cell growth arrest has been considered irreversible, while p53/p21 pathway-induced cell growth arrest is reversible [114]. Increasing levels of ROS can also activate p16, p53, and p21 via the MKK3/6-p38 MARK pathway [113].

Concurrently, increased levels of ROS may provoke SASP via the NFκB pathway, in addition to C/EBPβ, GATA4, and JNK transcription factors [122]. Dysfunctional mitochondria can also increase ROS/RNS and promote SASP [119]. The effects of mitochondrial dysfunction are aggravated because chondrocytes have reduced mitochondrial DNA content and reduced mitochondrial mass. Furthermore, the mitochondrial dysfunction occurs simultaneously with decreases in the expression of sirtuin 1 (SIRT1) and peroxisome proliferator–activated receptor γ coactivator 1α (PGC-1α), which are both implied in mitochondrial biogenesis, and the downregulation of the nuclear respiratory factors 1 and 2 (NRF1 and NRF2), which modulate antioxidant gene expression [122].

Reduced levels of NAD^+^ affect the functions of sirtuins (SIRT1–7), consequently inducing senescence via p53/p21. Alterations to the NAD^+^/sirtuin pathway can also negatively affect FOXO and PGC-1 functions, leading to increases in ROS and mitochondrial dysfunctions. FOXO transcription factors cause elevations in the expression of SOD2 and catalase. FOXO1/3 are mainly inhibited by AKT and the activation of AKT-initiated senescence due to the augmentation of intracellular ROS levels. The FOXO4 activated by JNK provokes senescence by activating the p21 pathway. 

Endoplasmic reticulum (ER) stress caused by a redox imbalance can also induce chondrocyte senescence. ER stress downregulates the expression of collagen type II and aggrecan in the cartilage matrix and increases chondrocyte apoptosis [120].

Finally, among regulatory ncRNAs, which are highly responsive to specific stimuli, including those evoked by oxidative stress, miRNAs, lncRNAs and, more recently, circRNAs have been shown to play important roles in the pathophysiology of cellular senescence [119,123].

## 5. Protection of Chondrocytes against Inflammation

### 5.1. Chondroitin Sulfate and Glucosamine

As neutrophils are not present in OA synovial fluid, this disease was long considered a non-inflammatory arthropathy. This picture began to change when the involvement of pro-inflammatory cytokines, such as IL-1β and TNF-α, was clearly recognized in the degradation of the articular cartilage ECM [124,125].

The cartilage ECM is very rich in chondroitin sulfate (CS). Most cartilage CS is covalently linked to the aggrecan core protein, and many aggrecan monomers interact with a hyaluronan molecule to form large complexes that provide the cartilage with a hydrated structure that furnishes the cartilage with its compressive stiffness and load-bearing properties. When the cartilage ECM is digested, the aggrecan core protein is degraded by ADAMTSs, and CS chains are released. These polymers appear in urine [126].

In 1999, CS was proposed as a dietary supplement for the management of OA symptoms [127], but its effectiveness was controversial and questioned by many authors [128]. One of the reasons that led to these divergent results is that different CS preparations, produced by different manufacturers, have different qualities. For instance, it was shown in 2015 that only five out of sixteen pharmaceutical-grade CS preparations from different countries really contained >95% CS [129]. Nevertheless, it was also observed that the treatment of mild OA in horses with CS, followed by glucosamine, led to a long-lasting and significant improvements in OA symptoms, including flexion test, lameness, and pain [126]. Although similar results have also been reported in humans (review in [130]), other authors also reported different results in humans [131].

For these reasons, CS and glucosamine became therapeutic alternatives for improving function and preventing pain in OA and were considered symptomatic, slow-acting drugs on OA (SySADOA) or symptom-modifying OA drugs (SMOAD) [132]. There is evidence that CS and glucosamine may also preserve and even repair damaged cartilage in OA in both humans [6,7] and animals [8]. It was shown that different CSs, irrespective of their origins, molecular weights, and structures, have anti-inflammatory actions on both chondrocytes and macrophages in culture, although with different efficacies. Chondrocytes were challenged with IL-1β, and macrophages were challenged with LPS. The effects of CSs on the production of TNF-α, IL-1β, IL-6, NO, and PGE2 were analyzed. CS had anti-inflammatory effects on both chondrocytes and on macrophages and hindered the nuclear translocation of the NFκB proteins [133]. It is possible that in vivo, the combined actions of CS on these different cell types may help its anti-inflammatory actions.

### 5.2. Other Agents

Collagen has been cited as an emerging research focus for joint health. [134,135]. Different collagen-derived products can be obtained depending on the manufacturing process. They can have different compositions, structures, and properties such as insoluble or soluble native collagen, which maintains a triple helix structure, gelatin (denatured collagen), and hydrolyzed collagen (peptides/amino acids), which can be manufactured from different degrees of hydrolysis [135].

The combined use of native and hydrolyzed collagens can be justified by the differences in their mechanisms of action. Certain peptides from hydrolyzed collagen are absorbed and accumulated in the cartilage. In vitro studies have shown that they promote cartilage regeneration by inducing chondrogenic proliferation and differentiation, as well as through stimulating the synthesis of macromolecules in the ECM such as proteoglycans and collagen type II [134]. They may also change the activities of osteoblasts and osteoclasts. On the other hand, native collagen provokes an immune-mediated response known as oral tolerance, a mechanism completely different from the mechanism described for hydrolyzed collagens. Collagen type II has been demonstrated to be a potential source of autoantigens in OA, and native collagen type II might reduce autoimmune reactions against endogenous collagen at the joint cartilage level [135]. The oral tolerance mechanism also allows for the secretion of anti-inflammatory mediators, including TGF-β, IL-4, and IL-10, which aid in decreasing joint inflammation and promoting cartilage repair [136]. Collagen has been found to reduce oxidative stress in chondrocytes, but the exact mechanism has not been fully elucidated [134].

Hyaluronic acid (HA) injections are often used in the treatment of OA, both in humans and in horses. HA is a linear, non-sulfated glycosaminoglycan that occurs in all animal tissues as a free polymer. It is synthesized by hyaluronan synthases (HAS1-3) at the cell surface which use cytoplasmic UDP-glucuronic acid and UDP-N-acetylglucosamine as substrates. HAS3 synthesizes HA with a smaller molecular mass than those synthesized by HAS1 and HAS2 [137]. HAS2 is much more active than HAS1, but HAS1 is induced by pro-inflammatory factors like interleukins and cytokines.

HA, although it does not form proteoglycans, can bind proteins on both the cell surface or secreted into the ECM. The major family of proteoglycans that bind to HA are hyalectans: aggrecan in cartilage, and versican, neurocan, and brevican in other tissues. These proteoglycans share homologous G1 domains that mediate the interaction with HA and form aggregates, as previously discussed (see Section 1). HA is catabolized by two different mechanisms: one is mediated by hyaluronidases, while the other is mediated by ROS, and the molecular weight of HA in cartilage is reported to decrease with age [138].

High-molecular-weight HA confers the synovial fluid its viscosity. In horses, the anti-inflammatory, antioxidant, and chondroprotective activities of low- and high-molecular-weight HA (LMW-HA and HMW-HA, 40 kDa and 1350 kDa, respectively) were compared to triamcinolone acetonide, the agent most frequently used to treat synovitis. Although it alleviated the symptoms, triamcinolone induced the breakdown of articular cartilage aggrecan and synovial fluid HA. In contrast, both LMW-HA and HMW-HA preserved the joints. Furthermore, HA also had anti-inflammatory actions, and LMW-HA was the most effective in reducing the release of cytokines [139].

As previously mentioned, HA also acts as a signaling molecule, binding with a high affinity to cell surface receptors named hyaladherins. In chondrocytes, three cell surface receptors that bind HA were recognized: CD44, receptor for hyaluronate-mediated motility (RHAMM), and intercellular adhesion molecule *1* (ICAM-1) [140]. Although the interactions of HA with RHAMM and ICAM-1 may trigger a variety of signaling pathways leading to HA’s anti-inflammatory actions, CD44 is the most important HA receptor in chondrocytes [138]. HA has been shown to reduce chondrocyte apoptosis while increasing chondrocyte proliferation via CD44 binding. The HA actions mediated by CD44 have been recently reviewed [138,140].

Finally, a few compounds extracted from plants were recently described to have joint anti-inflammatory and chondrocyte-protective actions. Two such compounds are curcumin, found in turmeric [141], and resveratrol (3,4′,5-trihydroxystilbene), found in red grapes, berries, and mulberries [142]. Curcumin was shown to have anti-inflammatory properties by inhibiting the TLR4 pathway and its downstream NFκB signaling pathway [140], while it was shown that resveratrol has a chondroprotective effect by significantly suppressing AGE-induced MMPs and increasing the expression of glycosaminoglycans through AP-1 and NFκB signaling pathways (review in [142]).

*Luzula sylvatica* was recently described as an anti-inflammatory plant, probably because its extract (LS-E) can decrease the production of ROS and may inhibit the secretion of PGE2 due to an inhibition of the expression and activity of COX-2. It was also observed that the EP4 protein, one of the receptors for PGE2, is decreased in the presence of LS-E. Therefore, LS-E might decrease joint inflammation related to PGE2/EP4 signaling, consequently interrupting the progression of OA. Furthermore, *Luzula sylvatica* can act directly on chondrocytes by diminishing the expression of proteases MMP-1 and ADAMTS4 [143].

## 6. Conclusions

In recent years, much has been learned about the biology of chondrocytes and cartilage: the master control genes for chondrocyte differentiation, the importance of chromatin organization for chondrocyte homeostasis, and the mechanisms of chondrocyte signaling, autophagy, oxidative stress, and death have been studied and elucidated. There are still many points to be clarified, such as the role of extracellular vesicles in the development and evolution of OA, the functions of ncRNAs in normal chondrocytes, and the changes that occur in diseases—are they a cause or a consequence of the disease? It is possible that soon, all this knowledge will lead to the development of new drugs and therapies that permit the early diagnosis, treatment, and control of OA and other joint diseases.

## Data Availability

Not applicable.
